# The effectiveness of postpartum interventions aimed at improving women’s mental health after medical complications of pregnancy: a systematic review and meta-analysis

**DOI:** 10.1186/s12884-022-05084-1

**Published:** 2022-11-03

**Authors:** Jie Shang, Nadila Dolikun, Xuanchen Tao, Puhong Zhang, Mark Woodward, Maree L. Hackett, Amanda Henry

**Affiliations:** 1 Discipline of Women’s Health, School of Clinical Medicine, UNSW Medicine and Health, Sydney, Australia; 2 The George Institute for Global Health, Beijing, China; 3 The George Institute for Global Health, Faculty of Medicine and Health, University of New South Wales, Sydney, Australia; 4 The George Institute for Global Health, School of Public Health, Imperial College London, London, UK; 5 Department of Women’s and Children’s Health, St George Hospital, Kogarah, NSW Australia

**Keywords:** Pregnancy complication(s), Postpartum mental health, Postpartum depression, Postpartum anxiety, Randomised controlled trial, Gestational diabetes mellitus, Preeclampsia, Hypertensive disorders of pregnancy

## Abstract

**Background:**

Postpartum mental disorders including depression and anxiety are common. Medical complications of pregnancy, such as preeclampsia and gestational diabetes, are thought to increase the risk of mental disorders postpartum. However, it is unclear which interventions may be effective for preventing and/or treating postpartum mental disorders following a medically complicated pregnancy. We aimed to systematically review published literature on the effectiveness of postpartum interventions to improve women’s mental health after medical complications of pregnancy.

**Methods:**

Systematic review (PROSPERO: CRD42021220030) was performed. Eligibility criteria: (1) randomized controlled trials (RCTs), published 1st Jan 2001-12th August 2021 (2) outcome measures reported on postpartum mental disorders (3) participants had ≥ 1 medical complication during pregnancy (4) intervention entirely postpartum or contained a postpartum component (5) full-text available in English or Chinese. Risk of bias was assessed using the Revised Cochrane Criteria Risk of Bias. Random effects inverse-variance weighted meta-analysis was used to pool the individual standardized mean differences (SMD) in depression or anxiety scores between intervention and control groups.

**Results:**

Of 5928 studies screened, 9 met inclusion criteria, and were based on non-pharmaceutical, combined lifestyle interventions that began shortly after childbirth, or as part of extended care packages beginning during pregnancy. Of these, 2 were rated as low risk of bias, 1 with some concerns, and 6 were at high risk. Meta-analysis was performed for 8 studies using standardized measures of depression and 7 for anxiety. There were statistically significant reductions in depression (SMD − 1.48; 95%CI: -2.41 to -0.55), and anxiety scores (SMD − 1.98; 95%CI: -3.03 to -0.94) in intervention versus control groups. Considerable heterogeneity was noted for pooled depression (I^2^ = 97.9%, *p* < 0.05), and anxiety (I^2^ = 96.8%, *p* < 0.05) results.

**Conclusion:**

Limited intervention studies aimed at improving postpartum mental disorders after medically complicated pregnancy were found, most with a high risk of bias. There was some evidence to suggest that postpartum depression and anxiety scores improved after early intervention. However, in general the current quality of evidence is low. Further, high-quality, interventional research is required in this understudied field.

**Supplementary Information:**

The online version contains supplementary material available at 10.1186/s12884-022-05084-1.

## Introduction

The postpartum period, especially within a few weeks to the first year after childbirth, is a critical stage in women’s lives in terms of mental health, with women vulnerable to onset or worsening of mood and stress disorders [1]. Diagnostic criteria for postpartum mental disorders, including postpartum depression and anxiety disorder, usually correspond to a certain time period after giving birth (up to 4 weeks post-delivery, although women remain at increased risk for several months after giving birth) [2, 3]. The worldwide prevalence of common postpartum mental disorders, including postpartum depression, anxiety, and post-traumatic stress disorder (PTSD), are estimated to be 17%, 9.9% and 4%, respectively [4–6]. Postpartum mental disorders are associated with several maternal and child adverse health outcomes, including poorer maternal quality of life and impaired infant development [7, 8].

Medical complications of pregnancy are those occurring when a woman’s body cannot adequately adapt to the sudden physiological changes due to gestation [9]. Common medical complications of pregnancy include gestational diabetes mellitus (GDM), affecting 1 in 7 pregnancies, and hypertensive disorders of pregnancy (HDP) including preeclampsia, affecting 5–10% of pregnant women [10, 11]. It is recognised that pregnancy complications can continue to affect maternal and child health after childbirth [12]. Apart from physical health consequences, postpartum mental disorders have also been associated with medically complicated pregnancy. Studies have reported up to 7 times higher risk of postpartum depression, more than 6 times elevated anxiety risk, and 5 times increased PTSD risk in women with preeclampsia, compared with that in normotensive women [13–16].

Interventional studies are plentiful regarding mental disorder prevention and management for postpartum women in general, including early detection and screening, as well as treatment based on various strategies [17, 18]. A 2015 systematic review of 45 RCTs found that 37 (80%) used psychological interventions, such as interpersonal therapy (IPT) and cognitive behavioural therapy (CBT), to prevent postpartum depression. The remainder used pharmacological methods, antidepressant drugs or micronutrients, such as omega-3 fatty acids and dietary calcium [18]. Of the 45 RCTs, 20 (45%) found their intervention to be effective in depression prevention, with the rest finding no effect [18]. In another review of RCTs aimed at general postpartum support, researchers found no evidence that general provision of postpartum support can improve any of the outcomes studied, including parenting, maternal mental health or quality of life. However, “high-risk” women, with family dysfunction or abuse, were found to benefit from a home-visiting based support intervention, and the home environment quality, family function, as well as peer support, were improved [19].

Despite the extensive literature around postpartum mental disorder prevention and control generally, the evidence regarding early postpartum interventional studies for women after a medically complicated pregnancy is unclear. Therefore, the aim of this research was to systematically review academic literature in the past 20 years and evaluate the effectiveness of early postpartum care interventions to improve mental health for women after medical complications during pregnancy.

## Methods

A systematic review was conducted according to Preferred Reporting Items for Systematic Review and Meta-analysis (PRISMA) guidelines [20]. The systematic review was registered in the PROSPERO registry (CRD42021220030).

### Eligibility criteria

The inclusion criteria were: (1) RCTs, (2) postpartum mental health outcomes (e.g., number of cases of a specific disorder after intervention, or changes in symptom scores on quantitative measures of a specific disorder between baseline and primary endpoint); (3) participants were pregnant or postpartum women within one year of childbirth, who had at least one medical complication (e.g. hypertensive disorder of pregnancy, gestational diabetes) diagnosed during pregnancy; (4) intervention conducted during the postpartum period, or at a minimum contained a postpartum component; (5) full-text available written in English or Chinese.

### Search strategies

The following electronic databases were searched: MEDLINE, EMBASE, Cochrane Central Register for Clinical Trials, Global Health, and PsycINFO. Additionally, the two most relevant Chinese databases, China National Knowledge Infrastructure (CNKI) and Wan Fang, were also searched. (These two Chinese databases are ranked top 2 regarding number of journals, core journals and full-text coverage, as well as other resources including theses and dissertations) [21]. Combinations of related terms and equivalent subject were applied for “pregnancy complications”, and “postpartum mental disorders” (See Supplementary File 1. for details of search strategies and results). Searches were limited to studies with available abstracts published from 2001 through to 12th August 2021, to improve confidence that trial methodology and patient characteristics would be similar enough to those of the present day to be relevant.

### Study selection

Three reviewers (JS, ND and XT, public health researchers who are fluent in writing and reading English and Chinese), selected studies according to the eligibility criteria. Disagreement was resolved through discussion with senior researchers (AH for English-language studies, PZ for Chinese-language studies). Search results of full reference details from each database were imported into Endnote software for further selection [22].

### Data extraction

Data were extracted (by JS, ND and XT) independently using standardised forms. Extracted data included study setting, study population, demographics and baseline characteristics, details of the intervention and control conditions, study methods, recruitment and study completion rates and measures of outcomes. Disagreement was resolved through discussion with senior researchers (AH for English-language studies, PZ for Chinese-language studies). If study data were missing, an email or postal letter was sent to the study investigators requesting unreported data/additional details; please find details in Supplementary file 2. Only one author replied to the email [23], but no extra data were obtained.

### Quality assessment

Three researchers (JS, ND and XT) independently assessed the risk of bias of included studies using the Revised Cochrane Criteria Risk of Bias (RoB2), which includes assessment of randomization process, deviations from original intervention, outcome measurement, completeness of outcome data, and selective reporting [24]. Disagreements between the review authors over the risk of bias in individual studies was resolved by discussion, with involvement of a third author (AH for English language studies and PZ for Chinese language studies) where necessary.

### Data selection

For studies that used multiple measures to assess one specific mental health condition e.g., depression, data selection was based on following hierarchy to avoid meta-analysis of duplicate populations: (i) we selected data from the outcome measure that had been validated for use in postpartum women; (ii) if none of the measures had been validated, we selected the outcome measure used in the most included studies; (iii) if the number was equal, we selected the outcome measure with the largest number of participants (denominator). For example, in the study [25] reporting data from the Zung Self-rating Depression Scale (SDS) and Hamilton Rating Scale for Depression (HAM-D), whilst the primary outcome measure was unknown, we selected HAM-D scores because there was evidence of validity assessing depression in women during pregnancy and the postpartum period [26–30], not available for the SDS. Moreover, data were selected from the Hamilton Rating Scale for Anxiety (HAM-A) rather than the Zung Self-rating Anxiety Scale (SAS) due to the larger number of included studies reporting HAM-A scores [25, 31, 32].

### Statistical analysis

Random effects inverse-variance weighted meta-analysis was used to pool the individual standardized mean differences (SMD) in outcomes between the intervention and control groups using Stata/SE 17 software (commends including ‘metan’, ‘meta trimfill’ were performed for meta-analysis) [33, 34]. To assess heterogeneity between studies, I^2^ was calculated, and Cochran’s Q test applied. The statistical analysis script is attached as Supplementary File 3. We interpreted the amount of heterogeneity as low (0–29%), moderate (30–49%), substantial (50–89%), and considerable (90–100%) using I^2^ values following the Cochrane Handbook [35]. Funnel plot and Egger’s test was performed to assess whether publication bias existed [36, 37].

## Results

### Search results

**Fig. 1:**
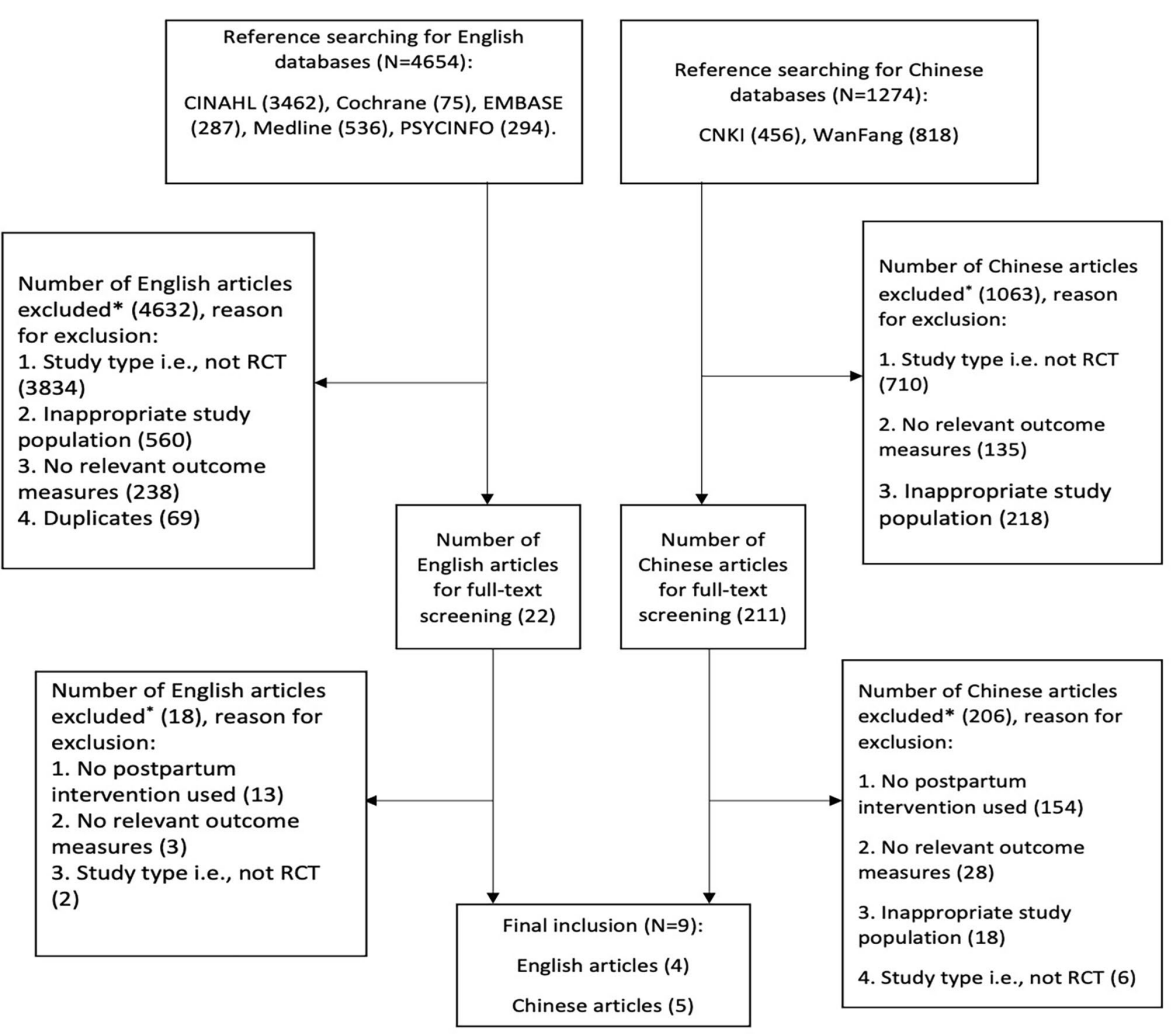
Flow chart for study selection procedures (The hierarchical exclusion follows that when studies have multiple reasons for exclusion only the first reason in the hierarchy is recorded)

As shown in Fig. 1, the electronic searches yielded 5928 references, including 4654 from English language databases and 1274 from Chinese databases. After title and abstract review, 211 references (22 English and 189 Chinese) remained for full-text screening. Finally, 9 RCTs were included, and these are summarised in Table 1.

**Table 1:** Characteristics of included studies

Study reference	Country	Medical condition	Participants	Interventions	Outcome measure	Outcomes
O’Reilly, S. L., et al. 2016 (38)	Australia	GDM	Control (n = 289), Intervention (n = 284);	*Intervention group*: lifestyle diabetes prevention intervention (MAGDA-DPP) includes 2 phases: Intensive phase (1 individual session, plus 5 group sessions on knowledge, skills of T2DM); maintenance phase (2 telephone sessions). *Control group*: usual care during RCT, and intervention after 12 mo of final assessment.	PHQ-9^1^	*Control group*: baseline 4.57 (0.23), endpoint 4.39 (0.25), difference − 0.19 (0.449); *Intervention group*: baseline 4.06 (0.23), endpoint 4.41 (0.26), difference 0.35 (0.172). Between group difference baseline − 0.51 (0.111), endpoint 0.03 (0.943), *p* = 0.132.
Yu et al. 2017 (39)	China	GDM	Control (n = 33), intervention (n = 40).	*Intervention group*: combined care and management program during pregnancy (including mental health education, blood glucose monitoring and lifestyle management skills, one-to-one guidance), with postpartum telephone sessions every week until 2 months postpartum. *Control group*: usual care including health education, monitoring, and medication guidance.	SAS^2^, SDS^3^	SAS: *Intervention group*: baseline 55.15 (8.23), endpoint 43.27 (5.19), *control group*: baseline 56.65 (8.19), endpoint 51.39 (6.28), *p* < 0.05. SDS: *intervention group*: baseline 57.11 (8.27), endpoint 47.11 (2.19); *control group*: baseline 57.09 (8.49), endpoint 54.59 (3.72), *p* < 0.05.
Gerli et al. 2018 (42)	China	HDP	Control (n = 30), intervention (n = 30).	*Intervention group*: postpartum extended care: (1) organized extended care team consisting of experienced obstetricians and registered nurses, (2) individualized guidance at end of hospitalization, (3) online communication support (WeChat group for Q&A), and (4) home visit or telephone review. *Control group*: usual care including physical indicator monitoring, medication guidance, pain regulation, and relevant health education.	HAM-A^4^, HAM-D^5^	HAM-A: *Intervention group* (endpoint): 6.47 (1.05), *Control group* (endpoint): 10.45 (1.55), t = 11.64, P < 0.01; HAM-D: *intervention group* (endpoint): 6.97 (1.14), *control group* (endpoint): 11.05 (1.67), t = 11.05, *p* < 0.01.
Yin et al. 2020 (25)	China	HDP	Control (n = 42), intervention (n = 42)	*Intervention group*: usual care plus 6 month extended care program: (1) organized extended care team consisted of health professionals to schedule and monitor program implementation, (2) set up postpartum communication, (3) online Q&A conducted by hospital staff (through WeChat). *Control group*: usual care including individualized rest environment, diet monitoring (salt, and nutrients), and health indicator monitoring during peripartum period.	SAS, SDS, HAM-A, HAM-D	*Intervention group*: SAS: baseline 66.52 (4.21), endpoint 39.45 (3.83); HAM-A: baseline 31.67 (4.67), endpoint 15.49 (3.48); SDS: baseline 64.83 (3.81), endpoint 51.48 (2.67); HAM-D: baseline 29.47 (3.62), endpoint 24.51 (4.59). *Control group*: SAS: baseline 66.19 (4.68), endpoint 42.56 (3.77), HAM-A: baseline 34.58 (4.29), endpoint 22.37 (2.86); SDS: baseline 64.72 (4.22), endpoint 48.63 (7.15), HAM-D: baseline 29.86 (3.11), endpoint 17.63 (4.82). *P* < 0.05 for between group differences regarding SAS, SDS, HAM-A and HAM-D.
Zhang et al. 2020 (41)	China	GDM	Control (n = 30), intervention (n = 30)	*Intervention group*: extended postpartum care: (1) early intervention postpartum, including GDM health education, BG testing, nutrition guidance. (2) extended intervention postpartum, including enhanced education on childrearing, recovery exercise, home visits or review (online communication). (3) extended management, including body weight, self-monitoring of BG. *Control group*: usual care during peripartum period.	HAM-A, HAM-D	HAM-A: baseline: *control group* 32.7 (3.71), *intervention group* 32.74 (3.53), t = 0.021, p = 0.492; endpoint: *control group* 29.42 (3.42), *intervention group* 17.65 (2.31), t = 15.607, p < 0.001. HAM-D: baseline, *control group* 43.94 (4.56), *intervention group* 43.91 (4.52), t = 0.026, p = 0.49; endpoint: *control group* 34.27 (4.37), *intervention group* 21.19 (3.24), t = 13.169, p < 0.001.
Liang et al. 2020 (43)	China	HDP	Control (n-40), intervention (n = 40)	mHealth extended care intervention: (1) health education, (2) guidance at end of hospitalization, (3) setup of WeChat group (weekly information feed, online Q&A, monitoring diary writing, and individualized assistance, (4) care provider written report at 42d, 1 mo, 3 mo and 6 mo postpartum for each patient.	SAS, SDS	SAS endpoint: *intervention group* 32.48 (2.55), *control group* 47.89 (9.23), t = 0.000, p < 0.01. SDS endpoint: *intervention group* 25.77 (3.79), *control group* 55.79 (10.84), t = 0.000, p < 0.001.
Parfenova (23) et al. 2020	Canada	HDP	Control (n = 56), Intervention (n = 57).	*Intervention group*: a two-page pamphlet on postpartum care knowledge (developed by a multidisciplinary team of health professionals) is used. *Control group*: usual care including explanations about the risks and recommendations for future pregnancy and health.	Self-developed questionnaire	Self-developed Questionnaire that contained questions on anxiety level using standard Likert scale: (1-not worried, 6-extremely worried). Global anxiety: intervention group: baseline 3.7 (1.0), endpoint 3.8 (1.0); control group: baseline 3.9 (1.1), endpoint 4.0 (1.0), p = 0.67.
Guo et al. 2021(40)	China	GDM	Control (n = 160), intervention (n = 160)	*Intervention group*: intensive Lifestyle Modification (ILSM): Six biweekly, face-to-face sessions and 5 biweekly phone sessions delivered by trained local health workers during the 3-month intervention. *Control group*: usual care following the clinical guidelines for patients with GDM, including same brochure for health education.	WHOQOL-BREF^6^	Perceived stress: ILSM group: baseline 22.75 (6.60), endpoint 24.18 (7.33); control group baseline 22.82 (6.85), endpoint 24.60 (5.47), *p* < 0.05. Psychological QoL: ILSM group: baseline 12.81(1.87), endpoint 14.24 (2.16); control group: baseline 13.75 (1.87), endpoint 14.26 (2.04), *p* < 0.05.
Pan et al. 2021(44)	China	HDP	Control (n = 35), Intervention (n-35)	*Intervention group*: comprehensive care during the perinatal period: (1) Prenatal care. (2) Delivery care. (3) Postpartum care, including everyday blood pressure measurement and post-surgery care. Companionship, especially from the husband, was strengthened to encourage and support mothers and help take care of newborns. *Control group*: usual care including medical education, regular pre- natal care, and medication application as pre- scribed.	HAM-A, HAM-D	HAM-A: Baseline: control 10.02 (1.49), intervention 9.69 (1.22); endpoint: control 7.95 (1.24), intervention 6.58 (1.01), *p* < 0.05. HAM-D: baseline: control 1.22 (1.97), intervention 10.94 (2.01); endpoint: control 8.83 (1.44), intervention 7.01 (1.38), *p* < 0.05.

^1^ PHQ-9: Patient Health Questionnaire-9

^2^ SAS: Zung Self-Rating Anxiety Scale

^3^ SDS: Zung Self-Rating Depression Scale

^4^ HAM-A: Hamilton Anxiety Rating Scale

^5^ HAM-D: Hamilton Depression Rating Scale

The 9 RCTs included 1433 women participants who had medical conditions during pregnancy (Table 1). All 9 articles were published in the last 5 years (from 2016 to 2021). Four articles were published in English, and the remaining 5 were published in Chinese. Seven studies were conducted in mainland China, 1 in Australia and 1 in Canada. Participants from 4 studies were affected by GDM [38–41], and 5 by a confirmed diagnosis of HDP [23, 25, 42–44].

All 9 RCTs used non-pharmaceutical, combined lifestyle interventions that began shortly after childbirth, or as part of the extended care delivered from the prenatal period for the intervention groups. The control groups were described as being “routine care”. Researchers in 2 RCTs developed their own series of lifestyle modification interventions, composed of multiple sessions targeted at Type 2 Diabetes Mellitus prevention, the Mothers after Gestational Diabetes in Australia (MAGDA) intervention and Intensive Lifestyle Modification (ILSM) program [38, 40]. The content of those sessions covered knowledge on future disease risks, diet and exercise suggestions, as well as stress management. One study team developed a two-page pamphlet of educational materials on HDP adapted for plain language [23]. Three RCTs included mHealth based components in the postpartum interventions with smartphones used as an essential tool for intervention delivery, including timely communication between health professionals and participants [25, 38, 42, 43, 45]. Six RCTs delivered in mainland China adopted interventions called “extended” care programs, which means for a prolonged period (usually from 2 to 6 months postpartum) as an extension to the care provided by health professionals from the hospital where they delivered their children in addition to routine care for the control group [25, 42, 43].

For mental health measurements, 7 RCTs used common standardized questionnaires for depression symptom assessment, including one study using the Patient Health Questionnaire (PHQ-9) [38], 3 studies using the SDS [25, 39, 43], and four using the HAM-D [25, 41, 42, 44] (Table 1). For anxiety assessment, 6 RCTs used standardized measurements, including, 4 studies using the HAM-A [25, 41, 42, 44], 3 using the SAS (including one study that administered SAS and HAM-A measures) [25, 39, 43], and 1 RCT using a self-developed questionnaire to rate postpartum anxiety level by a Likert scale (from 1-not worried to 6-extremely worried) [23]. Moreover, one RCT measured psychological health in general using The World Health Organization quality of Life Questionnaire (WHOQOL-BREF) [40, 46] (Table 1). All the studies rating depression and anxiety conditions of postpartum women reported results based on change in scores, not rates of clinically diagnosed cases of depression or anxiety.

In terms of risk of bias assessment, two studies were rated as having a low risk of bias [38, 40], one had some concerns [23], and six were at high risk of bias [25, 39, 41–44]. Of the nine included RCTs, five described sequence generation procedures (Table 2). Only two RCTs provided evidence of allocation concealment and blinding [23, 40], whilst two articles clearly reported their study as an open-label RCT [23, 38]. We therefore classified unclear bias for the other five RCTs.

**Table 2:**
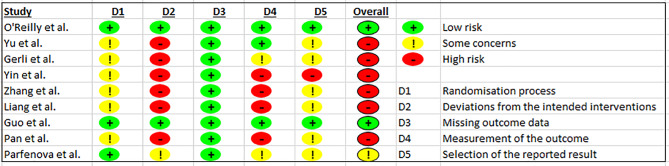
Results of risk of bias assessment using the Cochrane Criteria Risk of Bias Tool revised (RoB2)

^6^ WHOQOL-BREF: a shortened version of the WHOQOL-100 Questionnaire. It includes 26 questions and covers the physiological, psychological, social relations, and environmental dimensions.

In total, depression scores of eight studies were pooled, among which 4 studies measured postpartum depression using HAM-D [25, 41, 42, 44], 2 studies reported scores based on SDS [39, 43], one study used PHQ-9 [38], and one adopted WHOQOL-BREF that assessed psychological health in general, as shown in Table 3 [40]. Data from seven studies were pooled for anxiety, including 4 studies measuring postpartum anxiety using HAM-A [25, 41, 42, 44], 2 studies using SAS [39, 43], and the other using WHOQOL-BREF [40]. Overall, we observed more studies reporting greater reductions (fewer symptoms/less severe) in the scores for the intervention group than the control group, ranging from marginal to around 50% decrease, and over 40% lower mean scores for anxiety (Table 3).

**Table 3:** Results from studies reporting scores using depression and anxiety measurement scales

Study reference	Participants	N	Depression measure	Result of score at baseline (Mean (SD))	Result of score at endpoint (Mean (SD))	Anxiety measure	Result of score at baseline (Mean (SD))	Result of score at endpoint (Mean (SD))
O’Reilly et al. 2016	Intervention	284	PHQ-9	4.06 (0.23)	4.41 (0.26)			
	Control	289		4.57 (0.23)	4.39 (0.25)			
Yu et al. 2017	Intervention	40	SDS	57.11 (8.27)	47.11 (2.19)^2^	SAS	55.15 (8.23)	43.27 (5.19)^2^
	Control	33		57.09 (8.49)	54.59 (3.72)		56.65 (8.19)	51.39 (6.28)
Gerli et al. 2018	Intervention	30	HAM-D		6.97 (1.14)^2^	HAM-A		6.47 (1.05)^2^
	Control	30			11.05 (1.67)			10.45 (1.55)
Yin et al. 2020	Intervention	42	HAM-D	29.47 (3.62)	24.51 (4.59)	HAM-A	31.67 (4.67)	15.49 (3.48)^1,2^
	Control	42		29.86 (3.11)	17.63 (4.82)^1^		34.58 (4.29)	22.37 (2.86)^1^
	Intervention	42	SDS	64.83 (3.81)	51.48 (2.67)	SAS	66.52 (4.21)	39.45 (3.83) ^1,2^
	Control	42		64.72 (4.22)	68.63 (7.15)		66.19 (4.68)	42.56 (3.77) ^1^
Zhang et al. 2020	Intervention	30	HAM-D	43.91 (4.52)	21.19 (3.24)^2^	HAM-A	32.74 (3.53)	17.65 (2.31)^2^
	Control	30		43.94 (4.56)	34.27 (4.37)		32.76 (3.71)	29.41 (3.42)
Liang et al. 2020	Intervention	40	SDS		25.77 (3.79)^2^	SAS		32.48 (2.55)^2^
	Control	40			55.79 (10.84)			47.89 (9.23)
Parfenova et al. 2020	Intervention	57				Self-generated questionnaire on global anxiety	3.7 (1.0)	3.8 (1.0)
	Control	56					3.9 (1.1)	4.1 (1.0)
Guo et al. 2021	Intervention	160	WHOQOL-BREF (Psychological QoL)^#^	12.81 (1.87)	14.20 (2.17)^1,2^			
	Control	160		13.75 (1.87)	14.46 (1.92)^1^			
Pan et al. 2021	Intervention	35	HAM-D	10.94 (2.01)	7.01 (1.38)^1,2^	HAM-A	9.62 (1.22)	6.58 (1.01)^1,2^
	Control	35		9.69 (1.22)	6.58 (1.01)^1^		10.02 (1.49)	7.95 (1.24)^1^

^#^ General psychological health aspect, not specified for depression or anxiety. For QoL scoring, higher scores represent for higher self-rated quality of life

^1^ P < 0.05 was the significance level for measurements over time

^2^ P < 0.05 was the significance level for measurement over group

**Fig. 2:**
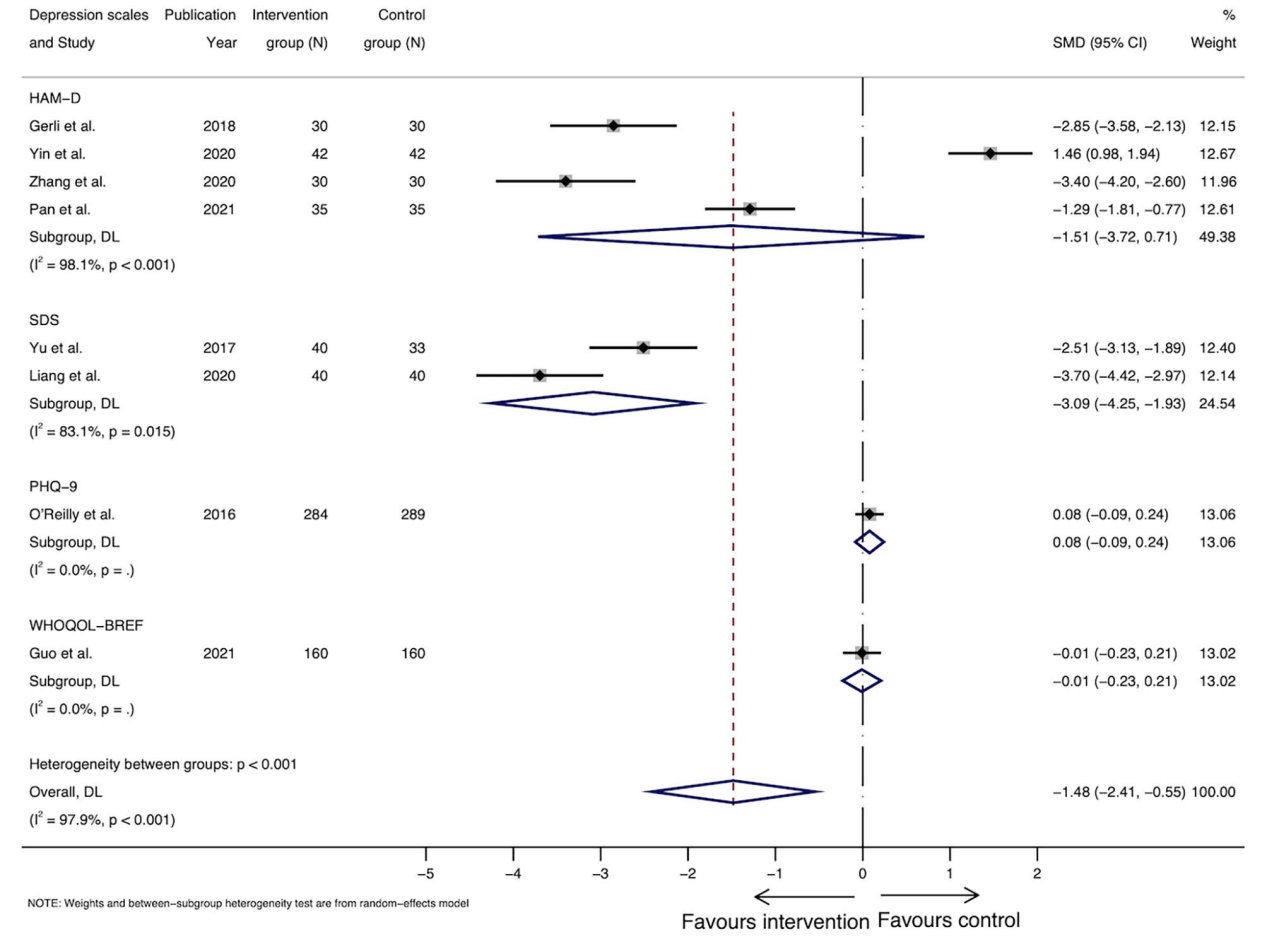
Forest plot of standardized mean differences in scores of depression measured at end of follow-up

**Fig. 3:**
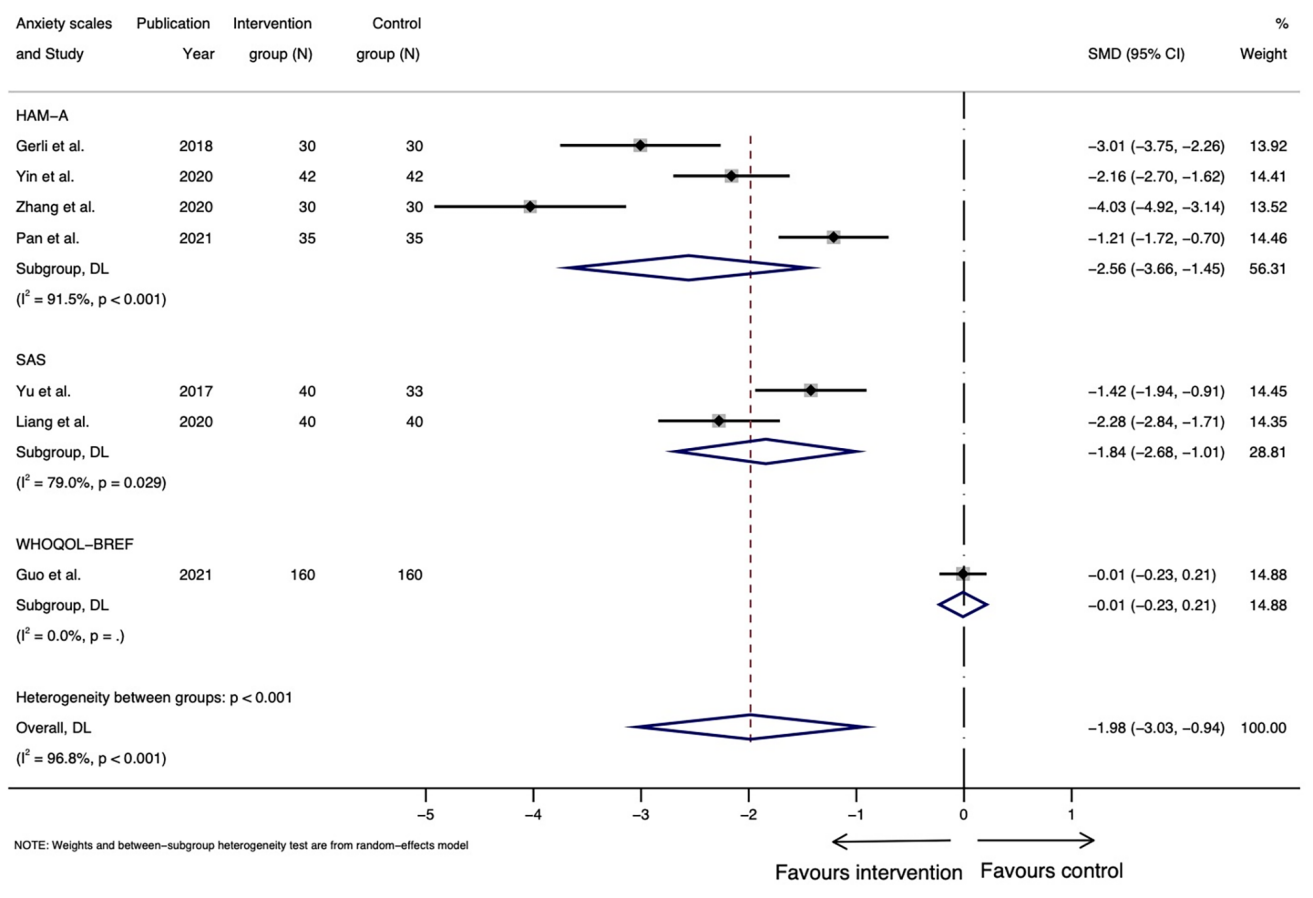
Forest plot of standardized mean differences in scores of anxiety measured at end of follow-up

**Fig. 4:**
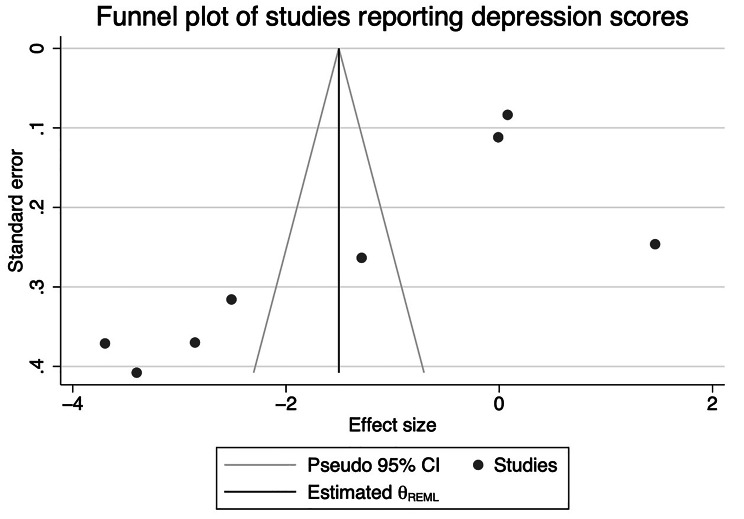
Funnel plot of studies included for meta-analysis of depression scores

**Fig. 5:**
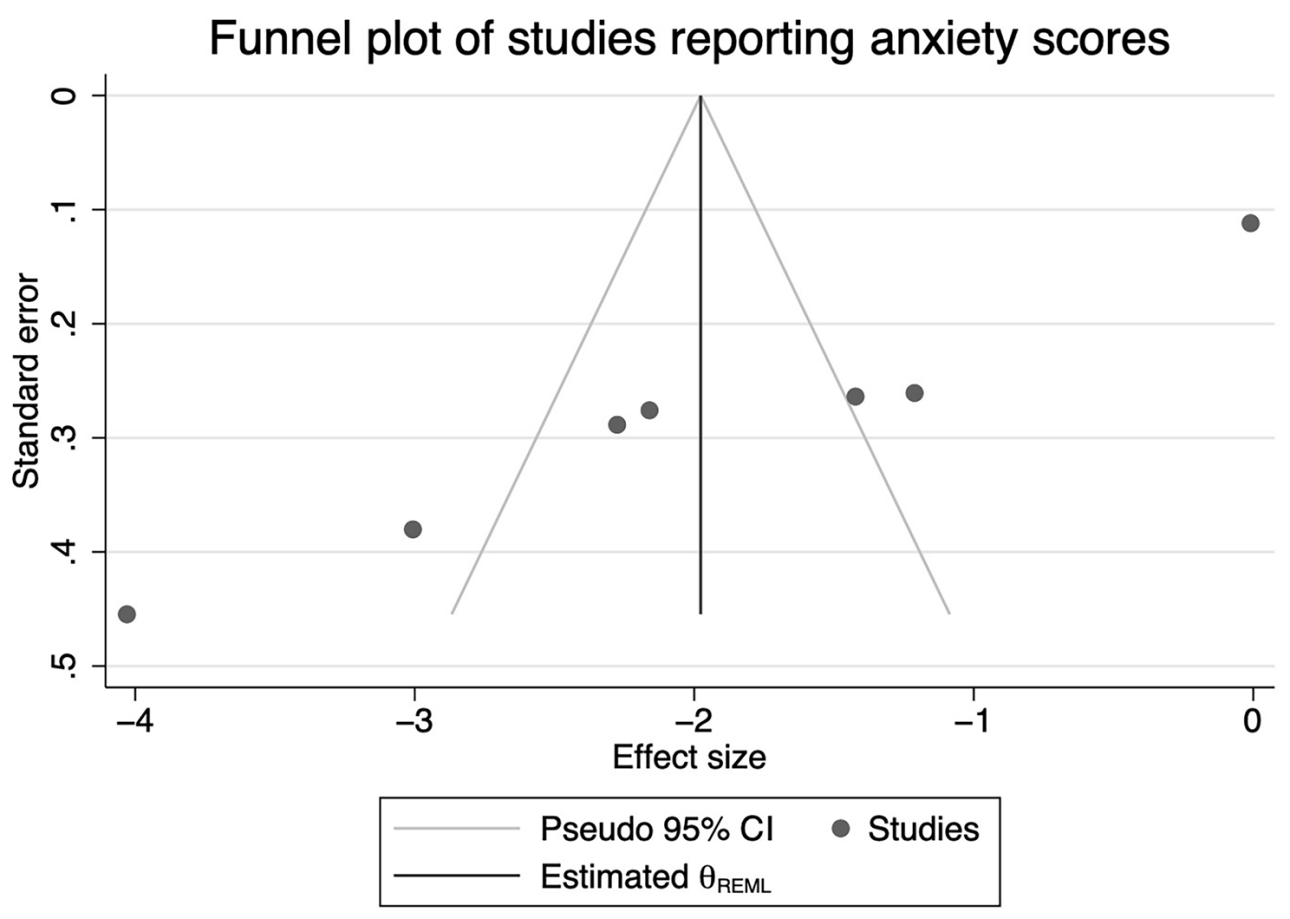
Funnel plot of studies included for meta-analysis of anxiety scores

Meta-analysis for postpartum depression was performed for the 8 RCTs, with 1320 participants, having comparable outcomes assessed by HAM-D, SDS, PHQ-9 and WHOQOL-BREF, as shown in Fig. 2. The overall standardised mean difference in scoring was − 1.48 (95% CI: -2.41 to -0.55) for the intervention group compared with the control group. However, considerable heterogeneity was noted (I^2^ = 97.9%, *p* < 0.001) [25]. For the 7 RCTs, including 747 participants, that were pooled for anxiety, the standardized mean difference in anxiety scores was − 1.98 (95%: -3.03 to -0.94) (Fig. 3), again with considerable heterogeneity (I^2^ = 96.8%, *p* < 0.001). Subgroup analysis and meta-regression were not performed due to the relatively small number of studies. Funnel plot was generated for studies reporting on depression (Fig. 4) and anxiety (Fig. 5) results, respectively. Asymmetry was found in the funnel plots of studies of both depression and anxiety scores and adjusted using ‘trim-and-fill’ method. However, no difference was detected after adjusting for the possible effect of small studies [37]. Results from Egger’s tests suggest that reporting bias is an issue for the depression (*p* = 0.03) and anxiety (*p* < 0.01) outcomes.

## Discussion

In this study, 9 RCTs meeting inclusion criteria were reviewed to determine the effectiveness of postpartum interventions on mental health outcomes among women diagnosed with medical conditions during pregnancy. Overall, these found that non-pharmaceutical interventions modestly reduced postpartum depression and anxiety symptoms after a complicated pregnancy. Our meta-analysis found that women’s depression (8 studies) and anxiety (7 studies) scores were significantly albeit modestly reduced by these interventions.

These findings are in line with interventional studies for postpartum mental health more generally. A review pooling results of 13 studies conducted in middle and low-income countries targeting common postpartum mental disorders, found that maternal depression symptoms can be improved by drug or non-drug (such as psycho-educational) interventions (SMD − 0.38, 95% CI: − 0.56 to − 0.21) [47]. Additionally, a recent meta-analysis shows that mHealth interventions, including telephone-based and smartphone app-based, can significantly decrease scores on the Edinburgh Postnatal Depression Scale (EPDS) (SMD= -1.09, 95% CI: -1.39 to -0.79) [48]. Another systematic review found a modest effect of depressive symptom relief after exercise-based interventions among postpartum women (SMD = − 0.64, 95% CI: − 0.96 to − 0.33), also measured by EPDS [49].Therefore, findings from this study’s targeted population of women after a medically complicated pregnancy is consistent with past literature for postpartum women overall.

RCTs included in this study are based on combined interventions covering multiple non-pharmacological strategies, including dietary and exercise guidance, blood pressure self-monitoring, breastfeeding as well as mHealth based telephone review and online “WeChat” discussion groups [41, 42]. The combined “extended” care program is a major characteristic of interventional studies conducted in China targeted at postpartum women [41, 42]. The “extended” care program is delivered by a professional team formed by obstetricians, nurses, nutritionists, and/or psychological health professionals from the hospital where women give birth. The program is considered a continuous process of nursing care during hospitalization. One possible reason is that women in China with common medical conditions, for example GDM, during pregnancy would experience a routine clinical pathway for health management during the prenatal period, which includes educational lectures, dietary and exercise guidance, and blood glucose monitoring [50]. Therefore, women might be more accepting of and adherent to a postpartum intervention as an extension of the prenatal care pathway.

Although we found a statistically significant overall improvement in anxiety and depression scoring in this study’s meta-analysis, the improvement cannot be directly interpreted as “clinically effective”. In another study reviewing the minimal clinically important change for common depression scales, the minimum improvement for HAM-D measurement was 28% (± 25.2%) for scoring of the 24-item full version, 27.1% (± 25.7%) for scoring of the 17-item version and 27% (± 25.1%) for scoring of the 21-iterm version [51]. For HAM-A scale, cut-off values are commonly used in the clinical environment to demarcate the various severity levels of anxiety, given that scoring 0–7 refers to no or minimal anxiety, 8–14 for mild anxiety, 15–23 for moderate anxiety, and 24 or higher for severe anxiety [52]. Therefore, merely decreasing scores cannot infer clinically important symptom alleviation. Results of this review only show a trend of reductions in depression or anxiety symptoms for women with pregnancy complications after some postpartum intervention.

Moreover, considerable heterogeneity was found in the depression and anxiety meta-analyses, suggesting high between-study variation [53]. For the forest plot of studies reporting depression scores (Fig. 2), results of three studies were divergent from the main trend of improved depression scores in the intervention group compared with the control group, which could be the major source of heterogeneity [25, 38, 40]. Reasons for the divergent trend of these study results may include the variety of interventions each study was based on [25, 38, 40]. In addition, psychological aspect was not the primary research question in most of the studies included, and the sample size was not calculated based on mental health measurements, which could result in failure to detect the true effect of those interventions on mental conditions of postpartum women. Therefore, the findings from the meta-analyses might not be generalisable to larger populations [54]. However, research has also found that a small number of studies included in meta-analyses can bias the heterogeneity (12–28% of I^2^ value for meta-analyses with a median number of 7 studies) [55]. Hence, the accuracy of the heterogeneity statistic may be compromised in this review.

One major limitation of this review is that the studies pooled were relatively small. Research suggests that small studies often result in greater heterogeneity compared with studies with large samples [56]. Ideal solutions to resolve high heterogeneity include sensitivity testing and subgroup analysis. However, another limitation of this review is that such solutions were not viable due to the small number of included studies. Moreover, only 2 out of the 9 eligible studies were of low risk of bias, raising further concerns about bias. Publication bias was also an issue among the included studies, although the pooled effect from meta-analyses remained the same after adjusting for possible bias. We expect that further high-quality studies, ideally using more common, validated perinatal measures such as the Edinburgh Perinatal Depression Scale, are needed on this research topic [57]. To maintain this systematic review up-to-date, we searched Cochrane Central Register of Controlled Trials for any trial registered between August 2020 to August 2022, but found no novel study on this research topic.

## Conclusion

In conclusion, this systematic review and meta-analysis has found some evidence that postpartum interventions combining health education and mHealth support can improve (reduce) anxiety and depression scores among women after a medically complicated pregnancy. Although a substantial amount of research has been conducted in women’s perinatal and postnatal mental health in general, women who were medically complicated during pregnancy are relatively less well studied. Further high-quality interventional research is required on this topic.

## Electronic supplementary material

Below is the link to the electronic supplementary material.

Supplementary Material 1

Supplementary Material 2

Supplementary Material 3

## Data Availability

The datasets analysed during the current study are available from the corresponding author on reasonable request.

## List of abbreviations

(PHQ-9): Patient Health Questionnaire-9
(SAS): Zung Self-Rating Anxiety Scale
(SDS): Zung Self-Rating Depression Scale. (HAM-A):Hamilton Anxiety Rating Scale
(HAM-D): Hamilton Depression Rating Scale
(WHOQOL-BREF): a shortened version of the WHOQOL-100 Questionnaire
(EPDS): Edinburgh Postnatal Depression Scale
